# Impact of Chemotherapy on Extracellular Vesicles: Understanding the Chemo-EVs

**DOI:** 10.3389/fonc.2019.01113

**Published:** 2019-11-19

**Authors:** Nur' Syahada Ab Razak, Nurul Syakima Ab Mutalib, M. Aiman Mohtar, Nadiah Abu

**Affiliations:** UKM Medical Center, UKM Medical Molecular Biology Institute (UMBI), Kuala Lumpur, Malaysia

**Keywords:** exosome, microvesicle, drug, therapy, cancer

## Abstract

Chemotherapy is the standard go-to treatment for cancer besides surgery and radiation. It has recently come to light that the interaction between chemotherapy and the immune system is important in maintaining tumor immunity as well as influencing the efficacy of the therapy. However, ample preclinical studies have shown that in addition to direct cytotoxic effects on cancer cells, a fraction of chemotherapeutic agents may promote immunogenic cell death, and alter the inflammatory milieu of the tumor microenvironment. Extracellular vesicles (EV) have been shown to interact with the tumor microenvironment by delivering alterative signals to the surrounding cells; as a result, this results in interference with each cell's capability to eradicate tumors or gives advantages to cancer cells so as to survive therapy. Chemotherapy-induced extracellular vesicles (chemo-EVs) have been theorized to be carrying different cargo loads than non-chemotherapy-induced EVs. Aside from chemoresistance, there is growing evidence to suggest that chemo-EVs could dictate tumor behavior, especially in terms of metastasis, immune response, and cancer stemness. This mini-review attempts to summarize and evaluate recent developments on the role of chemo-EVs in other aspects of tumor-related processes.

## Introduction

It is estimated that ~1,735,350 new cancer cases will be diagnosed in the United States per year, of which 609,640 people will die from these cancer-related diseases (1). The treatment for cancer varies depending on the stage, age of the patient, type of cancer, and presence of metastasis. New advanced treatments have been created and used to treat cancers, such as immune checkpoint blockade therapy and innovative surgical methods. Nevertheless, though advances in cancer treatment are promising, the usage of basic chemotherapy drugs to eradicate cancer is still commonly and widely used throughout the world.

The term chemotherapy was coined in the early 1900s by Paul Ehrlich, a famous German chemist, to describe any use of chemicals to treat any diseases (2). The introduction of chemotherapeutic drugs for the treatment of cancers dated back more than 50 years ago (3, 4). Since then, the invention and creation of other chemotherapeutic drugs skyrocketed into a multi-billion dollar industry (3, 5). The chemotherapy drugs are often a combination of multiple drugs or therapies, also termed polychemotherapy. These include several non-cytotoxic classes of therapy, such as hormonal therapy, and targeted therapy (biologic therapy) (6). There are multiple classes of chemotherapy drugs including taxanes, anthracyclines, anthraquinones, monoclonal antibodies platinum-based drugs, and others (7). For instance, platinum-based drugs such as cisplatin are often used to treat ovarian and testicular cancers (5), whereas taxane-based drugs such as paclitaxel are commonly used for the treatment of breast cancers (8). Chemotherapy regimen can be divided into several types depending on the administration. Neoadjuvant therapy is defined as any treatment therapy given before surgery or any local treatment inclusive of chemotherapy, radiation-based therapy, and immunotherapy (9). Adjuvant therapies, on the other hand, are treatment options given after the primary treatment to remove any remaining cancer cells (9). The modalities may include the usage of chemotherapy or a combination of that and radiotherapy or immunotherapy.

There has been a revolution in the medical oncology and pharmaceutical research arena as more drugs are becoming more effective and can be synthetically produced. Still, the most commonly used drugs to treat cancer nowadays are among the drugs that were discovered decades ago. For instance, cisplatin, one of the most widely used drugs and one of the earliest discovered compounds, is still effective at treating cancer today. Nevertheless, the relationship between chemotherapy drugs with tumor microenvironment is still elusive. To facilitate the efficacy of the treatment and understand the biology of post-chemotherapy, the association between chemotherapy drugs and its surroundings should be further apprehended. Interestingly, there have been multiple studies that demonstrate the link between drug treatments and the signaling of extracellular vesicles (EVs) (10–12). We therefore aim to review the biology of an emerging role of EVs, especially pertaining to the chemotherapy-induced extracellular vesicles (chemo-EVs), and discuss its physiological, and pathological roles in tumor biology.

## Heterogeneity of Extracellular Vesicles

EVs can be divided into several different types that include exosomes, apoptotic bodies and microvesicles (13, 14). Despite recent advances in understanding the EVs, the terms “exosome” and “microvesicle” have been used interchangeably in the literature (15). The heterogeneity of EVs has become one of the major limiting factors in understanding any EV-related biology (14, 16–19). The International Society of Extracellular Vesicles (ISEV) has recommended the general use of the word EVs when describing work related to exosomes and microvesicles unless specifically stated (14). The same type of cells may also release different types of EVs, thus increasing the complexity of EV-related biology (20). The biogenesis of EVs varies between different types of EVs and the mode of release (21). For instance, microvesicles are formed by the outward budding of the plasma membrane, whereas exosomes are released via the inward budding of the endosomal membrane, forming “multivesicular bodies” (MVBs) that will fuse with the plasma membrane, releasing exosomes extracellularly (21). The size of EVs are not fixed but rather dynamic. The size of microvesicles usually range between 50 and 500 nm while exosomes are between 50 and 150 nm (14). EVs were initially thought to be a mechanism for waste disposal by the cells, however, recent studies have shown that EVs alongside their cargos contain signals that allow cells to communicate with each other (22, 23). There are several potential mechanisms describing how the EVs are formed and how the cargos are sorted within the EVs (20, 22). However, this is a complex process and more studies are needed to comprehensively understand the biogenesis of EVs, especially the process of cargo sorting. This is because the process is very much dependent on different pathways that exists during the biogenesis of EVs as well as the state of the cells (20, 22). For instance, ubiquitination, SUMOlytion, and phosphorylation of cells have led to the different cargoes in EVs (20). This suggests that the biogenesis is influenced and regulated intracellularly depending on the need and readiness of the cells.

## Chemotherapy Induces Cancer Cells to Release Higher Amount of EVs

There are several reports showing that, upon chemotherapy treatment, cancer cells showed enhanced secretion of EVs (24, 25). For example, Lv et al. showed that certain drugs such as paclitaxel, irinotecan and carboplatin could significantly elevate the abundance of exosomes released from HepG2 (hepatocellular carcinoma cells) (26). The quantification of exosomes was achieved by measuring acetylcholinesterase (AchE) activity (26). AchE is an enzyme that is frequently used to quantify the level of EVs within a population (27). However, it is debated whether AchE can be used to monitor the overall levels of EVs or if, instead, a more robust method needs to be found (27). In a more recent study, Bandari et al. reported that in CAG human cells, when treated with drugs such as bortezomib, carfilzomib, and melphalan, the number of exosomes increased dramatically up until 16 h after treatment (24) as quantified by nanosight particle tracking analysis. Similarly, a study by Kreger et al. observed that when MDA-MB231 breast cancer cells were treated with paclitaxel, the number of exosomes shed by the cells increased as compared to the untreated control (28). This study also utilized the nanosight particle tracking analysis to quantify the number of exosomes. Nevertheless, most of the studies mentioned above were conducted in an *in vitro* setting using immortalized cell lines. The biology of EVs have been shown to be different between *in vitro* and *in vivo* and thus need further validation (29). Emam et al. used an *in-vivo* model in which Balb/C mice treated with doxorubicin produced a higher number of circulating exosomes in the blood (10). The exosomes were isolated using precipitation-based kits and the concentration of the exosomal proteins was measured. A similar study also reported that paclitaxel was able to induce a higher release of EVs in 4T1-bearing mice via nanoparticle tracking analysis (30). Moreover, in breast cancer patients, it was discovered that more EVs were secreted after post-neoadjuvant chemotherapy as compared to the basal levels (31).

However, there are some discrepancies in results with other studies. One study demonstrated that there was no significant increase in the number of EVs being released by ovarian cancer cells upon treatment with cisplatin (11). This study also quantified EVs using nanoparticle tracking analysis. However, the cells were only treated for 2 h prior to analysis, which may explain such a result. This also suggests that chemotherapy-induced EV may be cell type-specific and drug- or time-dependent. In another study, acute myeloid leukemia (AML) patients undergoing chemotherapy had a significant reduction in the exosomal protein concentration (32, 33). However, this study conducted the quantification of exosomes several days after chemotherapy induction. Ludwig et al. also showed similar results in which head and neck cancer patients that underwent oncological therapies had lower levels of exosomal proteins (34). We postulate that the burst in EV secretion following brief exposure of cells to cytotoxic drugs is likely short-lived because many of the tumor cells will undergo apoptosis and rapidly die, thus reducing the amount of released EVs. However, there is a possibility that the impact of the chemo-EVs could be substantial and lasting as no studies have reported on the time-limiting factor of EV secretion yet. The high release of EVs upon treatment with chemotherapy is most likely due to the cellular stress induced by the drugs. Similar to how cells release other types of damage-associated molecular patterns (DAMPs), such as uric acid and DNA, EVs that are released are also a response to damage induced by the chemotherapeutic drugs. It has been previously reported that exosomes can be released as DAMPs as a result of physical stress or local tissue damage (35). Moreover, based on these studies, it can be observed that the method for isolation and quantification of EVs varies from one group to another and must be considered when reporting the release of EVs. Certain methods may need complementary experiments to support the results; for instance, the AchE measurement is a more indirect method of EV quantification and may need further validation (27). Overall, based on the abovementioned studies, it can be suggested that chemotherapy may indeed induce the release of higher amounts of EVs, but further in-depth research is needed.

## Chemo-EVs Modulate Immune System/Response and Inflammation

Until today, research has shown that EVs play a role in modulating immune responses, including immune stimulation and immune suppression (26, 30). Programmed cell death-Ligand1 (PD-L1) is a classical immune surface protein that stops the anti-tumor function of T cells by binding to its receptor, programmed cell death-1 (PD-1), and effectively protects the tumor from immune surveillance (36). Del Re et al. first demonstrated that exosomal PD-L1 expression changes during treatment with anti-PD-1 antibodies in melanoma and head and neck cancers (37). Furthermore, the results showed that PD-L1 levels in plasma-derived exosomes significantly decreased in patients responding to treatment and increased in subjects with disease progression. In a similar fashion, Ludwig et al. (34) showed that in head and neck cancer patients with no active disease after completing oncological treatment the exosomes had lower PD-1 and PD-L1 expression (34). These studies show that cancer chemo-EV can influence the efficacy of other modes of intervention such as immune checkpoint blockade immunotherapy. Moreover, Lv et al. have shown that certain anti-cancer drugs, such as carboplatin and paclitaxel, can induce the release of exosomes containing heat shock proteins from HepG2 cells (26). These heat-shock protein-containing exosomes were shown to effectively induce natural killer cell cytotoxicity through the up-regulation of granzyme B. A similar outcome was achieved by Vulpis et al. in which they demonstrated that, upon exposure to melphalan (a genotoxic drug for multiple myeloma) the derived exosomes can stimulate the release of IFNγ by natural killer cells (38). Furthermore, a study by Lian et al. showed that the chemotherapy drug irinotecan induces a massive release of double-stranded DNA that can move into the cytosol of macrophages and dendritic cells and subsequently activate the inflammasome complex (12). Based on these studies, chemo-EVs have immunomodulatory effects and can influence other immune and inflammation players. It can be argued that the chemo-EVs are immunoactivators and can enhance the efficacy of the anti-tumor immune response in some cancers. However, this is not true for some cases. Zhang et al. showed that chemotherapy induced EVs can be immunosuppressive; chemotherapy induces the release of B-cell derived exosomes that can suppress cytotoxic T cells (39). They concluded that by decreasing CD19+ EVs, the potential of chemotherapeutic agents could be improved. This study focuses on EVs derived from immune-cells, whereas other studies report on cancer-derived EVs. The function of the EVs may be dependent on which cells are releasing the EVs as they may act differently even under the same stimuli.

## Chemo-EVs Carry Cargo With Pro-Tumorigenic Properties

Upon induction with chemotherapy, the contents within the released EVs may be altered as well, and this could influence the recipient cells. For instance, a study by Bandari et al. demonstrated that chemotherapy-induced EVs contained higher levels of heparanase on their surface (24). Heparanese is an enzyme that can degrade the surrounding extracellular matrix and be transferred intercellularly, resulting in altered tumor behavior (24). In a different study conducted by Kreger et al. breast cancer MDA-MB231 cells treated with the chemotherapeutic drug paclitaxel released exosomes enriched with surviving, a pro-survival protein (28). Survivin belongs to the group of inhibitors of apoptosis proteins (IAPs) and has been known to have elevated expression in many types of cancers (40). This cargo molecule was able to induce and send a cell survival signal to the surrounding breast cancer cells (28). The increased expression of survivin is well-correlated with disease progression and therapy resistance (40). Furthermore, a study by Keklikoglou et al. showed that chemotherapy-induced EVs have higher levels of annexin A6 in breast cancer patients undergoing neoadjuvant chemotherapy as compared to the pre-treatment levels (30). Annexin A6 is a calcium-dependent protein that is part of the conserved annexin family (41) whose expression is elevated in several cancers, namely skin, breast and cervical cancers (41). Annexin A6 promotes invasiveness and motility of breast cancer cells (41). In fact, the A6 derived from the chemo-EV was shown to stimulate the pulmonary endothelial cells to produce CCL2. This will in turn promote themonocyte cells to the pre-metastatic niche, therefore resulting in metastasis and vascularization (30). A study by Pavlyukov et al. has shown that when glioblastoma multiforme cells undergo apoptosis due to oncological treatment the released EVs (apoEVs) carry a different type of cargo than untreated EVs (42). The apoEVs are enriched in spliceosomes and will, in turn, promote therapy resistance and proliferation of surviving tumor cells as compared to the non-apoEVs (42).

Besides protein, EVs are also known to carry nucleic acids such as microRNA (miRNA), messenger RNA (mRNA) and non-coding RNAs. For example, one study demonstrated that when hepatocellular carcinoma cells were put under stress conditions using chemotherapy drugs such as doxorubicin and sorafenib, the expression of linc-VLDLR, a long non-coding RNA, increased (43). The linc-VLDLR is associated with the cellular response, particularly concerning chemotherapy treatment (43). Moreover, in a recent study by Shen et al. (44), they reported that chemotherapy-induced EVs release miRNAs that can promote breast cancer stemness (44). It was shown that breast cancer cells treated with chemotherapy drugs altered the miRNA-derived EV content, such as miR-9-5p and miR-195-5p, which in turn affects the transcription factor One Cut Homeobox 2 and, eventually, affects stemness-related genes such as NANOG, OCT4 and SOX2 (44). This will eventually lead to the higher adaptation of cancer cells in resisting therapy (44).

## EVs as Biomarkers for Chemotherapy-Current Updates

Multiple studies have shown that, in response to chemotherapy, the cargo within the EVs are sorted differently and, thus, specific cargo may be used as biomarkers to monitor chemotherapy response. For instance, EV containing annexin A3 in ovarian cancer patients could be used to predict platinum resistance (45). The levels of annexin A3 increase proportionately with drug use (45). In synovial sarcoma, it was shown that EV-derived miR761 can be used as a biomarker to predict resistance to pazopanib (46). With the advent of next-generation sequencing (NGS) technologies, more biomarkers are being discovered to predict chemoresistance. For instance, NGS was utilized to profile EV-derived miRNA in ovarian cancer patients that are resistant to platinum-based chemotherapeutics (47). A panel of miRNAs (miR-181a, miR-1908, miR-21, miR-486, and miR-223) was found to be selectively abundant in ovarian cancer patients resistant to platinum drugs (47). Furthermore, in a breast cancer cohort, a study by Rodriguez-Martinez et al. profiled the serum exosomal miRNA before, during and after neoadjuvant treatment (48). It was reported that exosomal miRNA-21 expression was higher in metastatic patients and was directly correlated with the size of the tumor (48). Besides chemoresistance, EVs have also been shown to predict other symptoms like cardiac injury. In doxorubicin-treated mice, the expression of isoforms of glycogen phosphorylase could be used to predict early cardiac injury (49).

## EVs in Hematological Malignancies

Most of the reported EV-related studies were conducted in solid tumors. Here, we will discuss the biology of EV in hematological malignancies. It was previously reported that patients diagnosed with chronic lymphocytic leukemia (CLL) released higher amounts of EVs as compared to healthy individuals (50). This study also showed that the plasma level of CD52+ EVs reduced significantly in post-therapy of CLL patients and could be further used as biomarkers for CLL progression (50). In a separate study, it was discovered that the level of serum-derived EVs containing CD19+ and CD37+ are significantly correlated with the tumor burden in CLL patients (51). Furthermore, the EV content in acute myeloid leukemia (AML) patients are correlated with disease progression and response to therapy (32, 52). Hong et al. showed that post-induction chemotherapy in AML patients had lower levels of exosomal protein and TGF-B1 (52). Moreover, a different study demonstrated that AML cells that were resistant to apoptosis after therapy released altered exosomal proteins as compared to apoptosis-sensitive cells (53). Recently, it was shown that blast-derived exosomes could also suppress hematopoiesis in AML (54). Additionally, bone marrow stromal cell (BMSC)-derived exosomes were shown to influence the progression of multiple myeloma cells (55). This study concluded that BMSC-derived exosomes increased cell survival, proliferation, migration, and induced drug resistance in multiple myeloma cells (55). It can be observed that EVs derived from blood-based cancers also have the same effects as in solid tumors, in which EVs encapsulate different content that promotes cancer.

## Limiting Factors and Future Directions on Chemo-EVs

**Figure 1:**
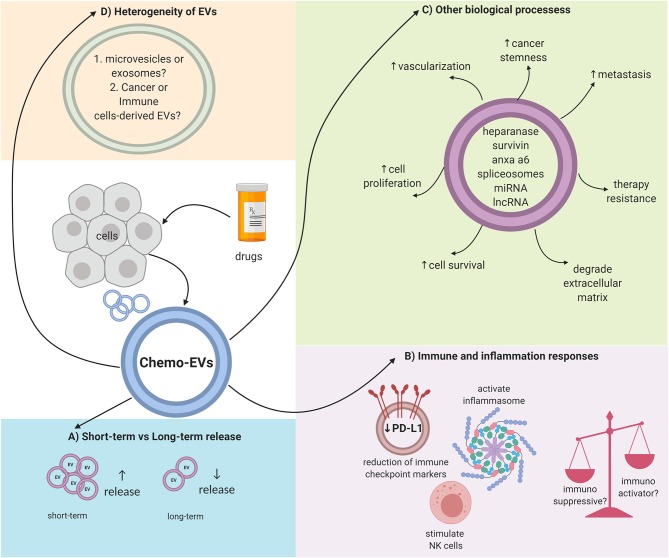
Overall schematic representation on the complex properties of chemo-EVs. **(A)** The number of released EVs could be dependent on the duration of drug stimulation. **(B)** Immunomodulating effects of chemo-EVs (e.g., reduced immune checkpoint markers such as PDL-1, activated inflammasome, and stimulate natural killer cells). Nevertheless, the uncertainty between the immunosuppressive and immunoactivator capabilities of chemo-EVs warrants further investigation. **(C)** Chemo-EVs affect other tumor biological processes such as cell proliferation and cell viability through the different cargoes that it carries. **(D)** The heterogeneity of chemo-EVs needs further elucidation to better understand the role of chemo-EVs.

EVs could be the future in medical research in terms of diagnosis or prognosis. EVs may be used as biomarkers, thus representing the ideal targeted cancer therapy approach contributing to the field of precision medicine. Nevertheless, there are still a lot of questions that need to be answered in response to chemo-EVs. First, the issue of heterogeneity within the EV population needs to be addressed (19, 23). EVs by themselves can be divided into several types, and whether these types overlap (in terms of cargo content or EV features) with each other should be determined. Moreover, within a specific subtype, the molecular composition can also be diverse (23). The mechanisms of how chemotherapy affects the heterogeneous composition of the EVs are far from understood. As discussed previously, certain chemotherapeutic drugs can induce a higher release of EVs while others may not, and, thus, further study is needed to link the induction of chemotherapy and the release of EVs. Furthermore, the techniques used to isolate and quantify EVs differ from one study to another. Further standardization is needed to truly evaluate the biogenesis and properties of EVs. Moreover, other physical properties of EVs should be addressed as well. For example, it is not known whether chemotherapy will affect the physical size or the surface charge/zeta potential of the released EVs. Furthermore, molecular markers expressed on the surface of EVs should be profiled as well-upon induction with chemotherapy drugs since the loss or gain of classical molecular markers of EVs has been reported as an effect of certain physical stress (56). Collectively, chemo-EVs can enhance our understanding on the biological effects of chemotherapy, especially in terms of the immune system, chemoresistance, and cancer progression. Figure 1 summarizes some of the key roles of chemo-EVs that have been reported. Overall, the effects of chemotherapy on EVs should be further investigated as they may play important roles in tipping the pro-and anti-tumorigenic balance in the tumor microenvironment.

## Author Contributions

NAR and NA drafted the manuscript. NA conceived the idea. NA, NAM, and MM provided the scientific input, critical feedback, and language editing.

### Conflict of Interest

The authors declare that the research was conducted in the absence of any commercial or financial relationships that could be construed as a potential conflict of interest.
